# A woman with disseminated tuberculosis experienced preterm delivery, fallopian tube pregnancy, and delivered successfully following in vitro fertilization: a case report

**DOI:** 10.1186/s12884-020-03487-6

**Published:** 2021-01-07

**Authors:** Ming Cheng, Tao Yuan, Ying Liu

**Affiliations:** Department of Reproductive Medicine, Beijing Obstetrics and Gynecology Hospital, Capital Medical University, Beijing, 100026 China

**Keywords:** Disseminated tuberculosis, Pregnancy, In vitro fertilization-embryo transfer (IVF-ET), Case report

## Abstract

**Background:**

Disseminated tuberculosis (TB) is a fatal disease resulting from hematogenous dissemination of *Mycobacterium tuberculosis*. Spontaneous pregnancy rate of women with TB is low; furthermore, live birth, spontaneous abortion or ectopic pregnancy may be the outcomes even if pregnancy occurs.

**Case presentation:**

We report a case of a woman with disseminated TB who had a series of complications including preterm delivery with congenital TB and infant death of pulmonary TB, fallopian tube pregnancy. She was treated by in vitro fertilization-embryo transfer (IVF-ET), and gave birth to a healthy baby.

**Conclusion:**

Disseminated TB has a significant impact on female fertility. We should take more active efforts to diagnose and treat this disease in a timely fashion. Moreover, IVF treatment is a feasible approach for an infertile woman after TB.

## Background

Disseminated tuberculosis (TB) is a fatal disease resulting from hematogenous dissemination of *Mycobacterium tuberculosis*. The diagnosis is challenging due to its non-specific clinical features, which is one of infectious causes of morbidity and mortality among women of childbearing age [1–3]. Unfortunately, spontaneous pregnancy rate is low, and if pregnancy occurs, live birth, spontaneous abortion or ectopic pregnancy may be the outcomes of women with pelvic TB [4, 5]. With the development of assisted reproductive technology, an increasing number of patients with infertility prefer to undergo fertility treatment by in vitro fertilization (IVF) [6].

In the present case report, we describe a successful treatment of a woman with disseminated TB through in vitro fertilization-embryo transfer (IVF-ET) following a series of TB-related complications including preterm delivery, infant death, fallopian tube pregnancy. Multiplie punctuate caseous lesions were adherent to the abdominopelvic cavity, which could also be seen on the surface of the bilateralfollopian tubes. We deem this case is significant both because of the unusual clinical presentation of disseminated TB in pregnancy and the histology-proven TB disseminated to the fallopian tubes, IVF-ET can also be a choice for a woman with disseminated TB.

To the best of our knowledge, rare cases of disseminated TB have been reported following IVF. Until now, there is no description about preterm delivery, fallopian tube pregnancy, and IVF occurring in a single patient.

## Case presentation

A 31-year-old woman, gravida 3, para 1, abortus 2, with a 2 years history of secondary infertility due to disseminated TB resorted to IVF for the therapy of her infertility. According to her past medical records, she was once diagnosed with the hematogenous disseminated TB and had a preterm birth and twice laparoscopic salpingectomy owing to fallopian tube pregnancy.

**Fig. 1:**
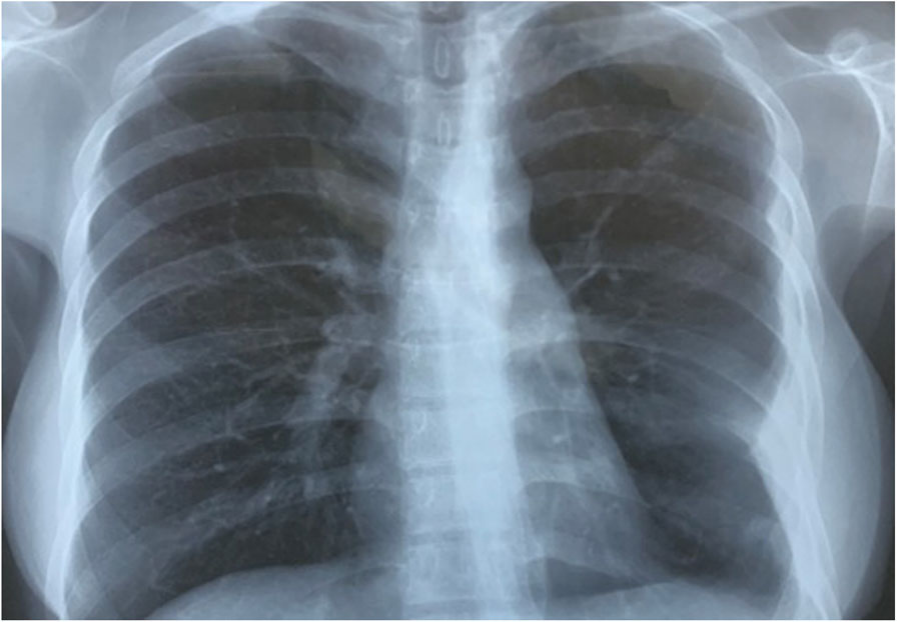
Posteroanterior chest radiograph showing miliary shadows in both lung fields

**Fig. 2:**
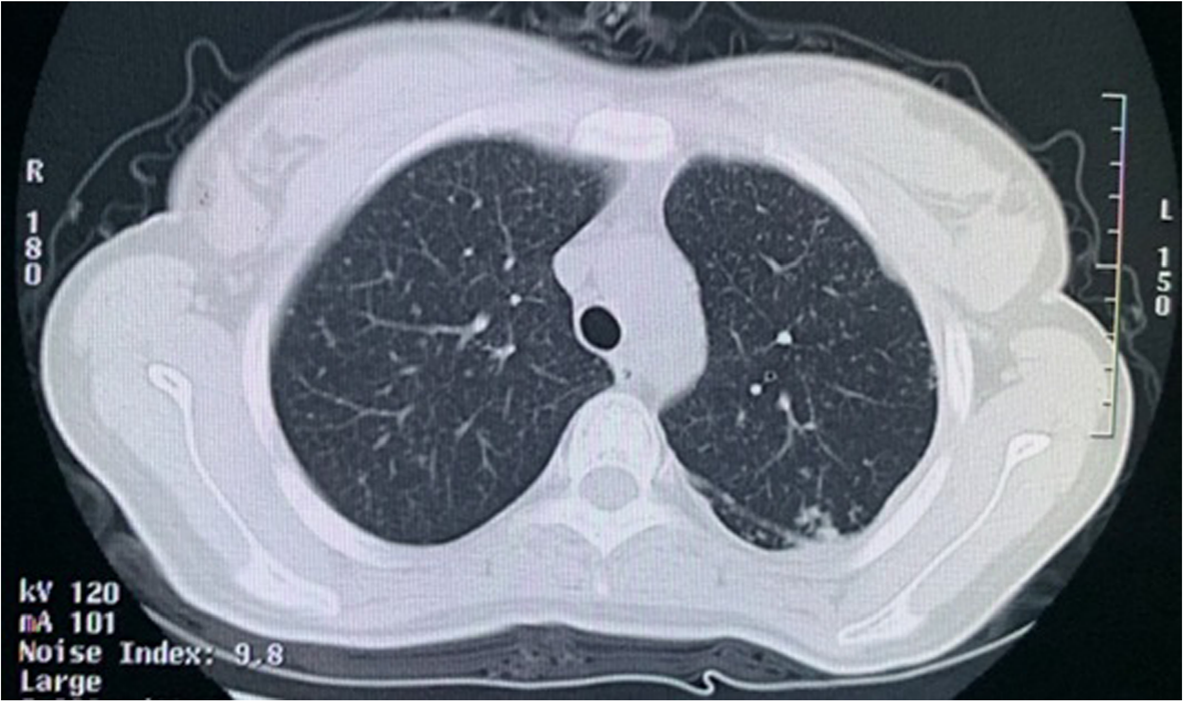
Representative chest computed tomography images showing multiple miliary nodules of uniform density, size and distribution

**Fig. 3:**
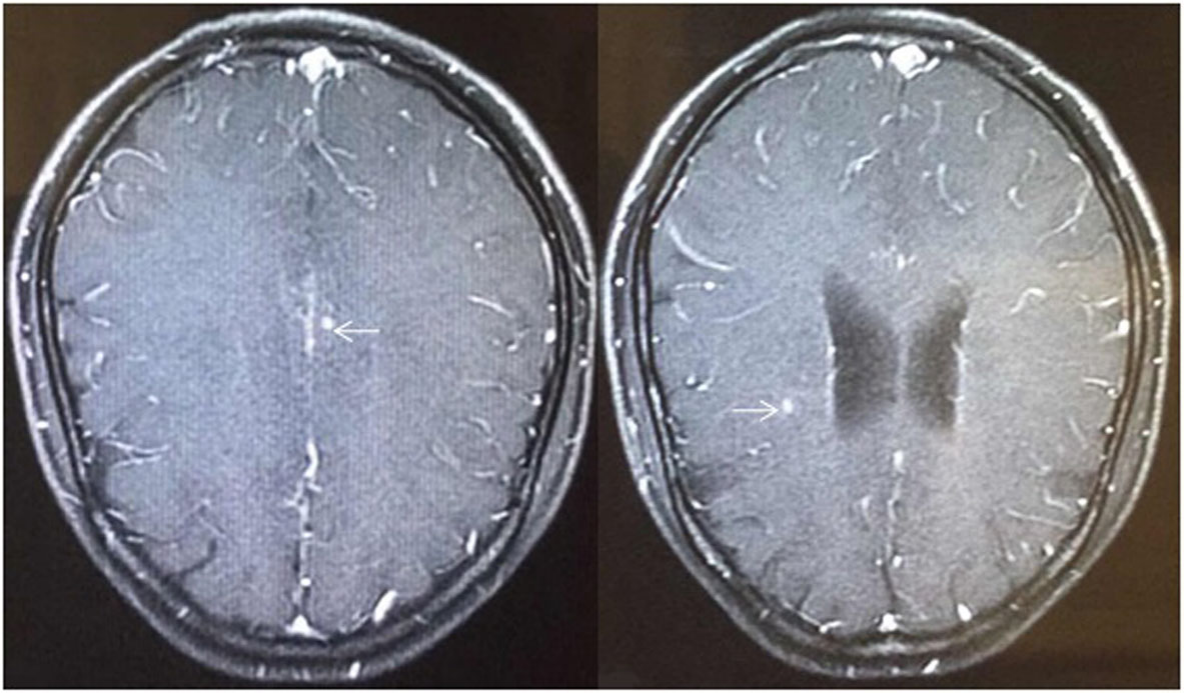
Cranial MRI showing bilateral centrum semiovale and multiple small plaques in the frontal lobe

In March of 2012, at her 28th gestational week, vaginal spotting occurred, and influenza-like symptoms appeared with intermittent low-grade fever. She was hospitalized due to uterine contractions followed by spontaneous rupture of the membranes, then she gave birth to a male infant by spontaneous vaginal delivery. The preterm neonate had congenital TB and died of “pulmonary tuberculosis” after 3 months. After the preterm delivery, her fever did not resolve. Her fever was persistent despite intravenous antibiotics administered for 1 month. The patient had a close family exposure tuberculosis contact history in the past, her father was infected by TB and her brother was diagnosed with tuberculosis meningitis. A chest X-ray was suggestive of TB (Fig. 1), prompting a computed tomography (CT) scan of the chest confirming the hematogenous disseminated TB with miliary nodular shadows throughout the bilateral lung parenchyma and left pleural effusions (Fig. 2). After the preterm delivery, she complained of nausea, vomit and severe headache. Her brain magnetic resonance imaging (MRI) showed bilateral centrum semiovale and multiple small plaques in the frontal lobe, consistent with an atypical infection such as TB (Fig. 3). A tuberculin skin test with purified protein derivative (PPD) was markedly positive at 9 × 11 mm.Then the patient continued the regular treatment of anti-tuberculosis for 2 years.

**Fig. 4:**
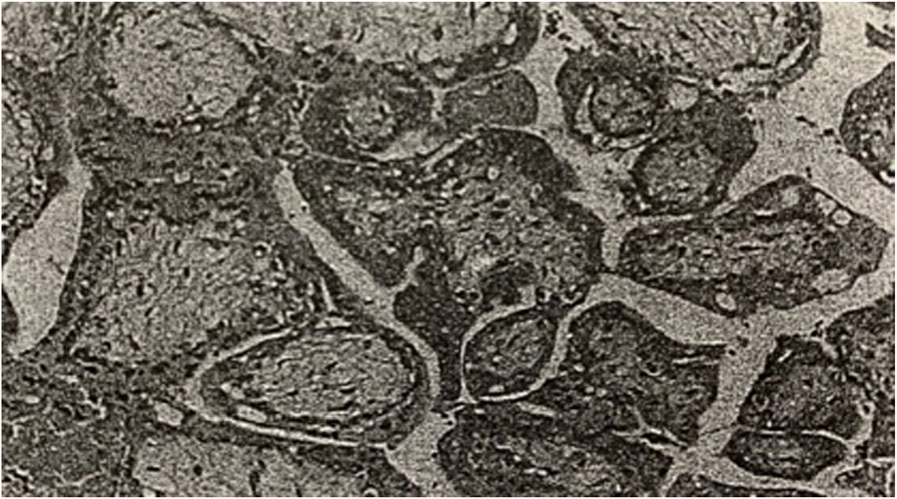
The pathology specimens of scraping content of uterine cavity showing interstitial decidua-like changes in endometrial hypersecretory response, with positive A-S sign of gland (HE × 10)

In July of 2015, the patient was sent to the emergency department and a laparoscopic salpingostomy was performed due to “left ampullary fallopian tube pregnancy”. During surgery, multiple punctate caseous lesions were pertinaciously adherent to the abdominopelvic cavity which could not be detached, and they could also be seen on the surface of the left tubal ampullary region. She underwent pelvic adhesiolysis. Biopsies of endometrium and the ampulla of left fallopian tube were performed. The pathology specimens of scraping content of uterine cavity showed interstitial decidua-like changes in endometrial hypersecretory response, A-S sign of gland is positive (Fig. 4). The left fallopian tube showed coagulation and villi tissue were approximately 1 cm in diameter, and micro-embryonic tissue can be seen. After the operation, the patient was diagnosed with “pelvic tuberculosis” without anti-tubeculosis treatment.

**Fig. 5:**
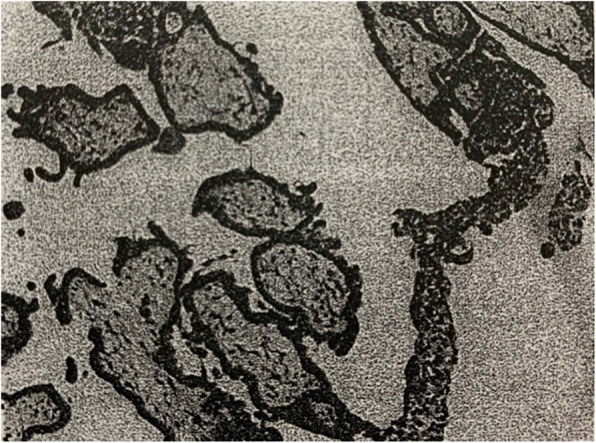
Biopsy of fimbria of right fallopian tube showing villi tissue

In November of 2016, the patient was hospitalized and an emergent laparoscopic salpingostomy was performed due to “right fallopian tube pregnancy”. Biopsy of fimbria of right fallopian tube was also performed showing 0.5 cm villi tissue (Fig. 5).

Upon presentation to our clinic, the patient’s hormonal level was normal and B-mode ultrasonography recordings indicated regular ovulations. Her husband’s semen analysis was normal. The patient’s menarche occurred when she was 12 years old, and her menses recurred every one month, lasting for 5 to 6 days. Recruitment of follicles was achieved by using the GnRH-antagonist protocol. She was stimulated by 150 IU/day unit of Gonal-F from the menstrual day 3 to day 4, and the dose was adjusted according to the follicular response. The amount of Gonal-F was increased to 225 IU/day from the menstrual day 5 to day 11, 0.25 mg/day unit of cetrotide was used from day 8 to day 12, and 75 IU/day unit of human menopausal gonadotropin (HMG) was added to improve follicular growth from day 10 to day 11. Trilaminar endometrium has been detected to be 10 mm thick and two dominant follicles greater than 17–18 mm diameter on hCG day. The patient had an outstanding estradiol response, and 22 oocytes were retrieved 36 h after the hCG injection by ultrasonically guided transvaginal approach and were inseminated with the husband’s semen. After 6 days, 8 blastocysts were frozen in order to avoid ovarian hyperstimulation syndrome (OHSS). Two months later, 2 embryos were transferred into the uterus after ovulation from natural menstrual cycle. Following embryo transfer, the patient received progesterone luteal support, which included oral progesterone capsules and vaginal administration of progesterone sustained-release vaginal gel. Then the patient conceived and ultrasonography revealed a single pregnancy by the presence of one gestational sac with living embryo. During the pregnancy, the patient did not have symptoms of TB recurrence. Ultimately, in January of 2020, the patient gave birth to a healthy baby girl through the natural childbirth after 36 weeks of pregnancy.

## Discussion and conclusions

It is easy for hematogenous disseminated TB to occur and spread in women of reproductive age since gestation and delivery are significant factors which can aggravate the potential TB. Mycobacterium tuberculosis bacilli can invade and attack the genital tract by hematogenous route and disseminate to foci outside the lungs. In most instances, gestation occurred when therapy implemented instantly after discovery of female genital TB, and when no irreversible anatomical pathology was obvious. However, having a successful uterine pregnancy is rare and the risk of ectopic pregnancy is high. In fallopian tube pregnancy, it is because of insufficient tissues and inadequate blood supply for placentation that makes most pregnancies abort in the early days. Besides, owing to limited expansibility and trophoblastic invasiveness in the tubal wall, rupture is extremely common, which can be life-threatening.

Multidrug anti-TB treatment is the backbone of therapy, but conception rates are not ideal amongst infertile women with genital TB even after multidrug anti-TB treatment. Caliskan [7] et al. had analyzed the effects of salpingectomy and antitubercular treatments on fertility results in patients with genital tuberculosis. Their results provided evidence that salpingectomy is a therapy option for patients diagnosed with tuberculosis and infertility due to the improvement of both clinical pregnancy rates and take-home baby rates. Meanwhile, although not statistically significant, patients should receive the antitubercular therapy for more than 6 months and ideally continue for 12 months. Therefore, it is essential for diagnosis that clinicians have a high clinical index of suspicion to some extent and take detailed history. Meanwhile, let patients take systematic inspections and a series of tests to document M. tuberculosis as well as imaging methods for characteristic structural changes [8]. Patients with a suspicious TB should undergo diagnostic workup like general clinical examination, ultrasonographic examination, and use of other diagnostic modalities to avoid diagnostic delay. Thus, early diagnosis followed by timely antitubercular therapy is required for optimal outcome, which is oftentimes still associated with poor prognosis in terms of the reproductive outcomes [9].

Women with TB can transmit mycobacterial tuberculosis through hematogenous placental to the fetus, and the fetus can ingest infected amniotic fluid or contact directly with the maternal genitals during birth. The former forms a major complex in the infant’s liver, and the latter a main focus in the lungs or gastro-intestine [10]. Besides, the possibility of postnatal transmission may exist when the infant’s mother wasn’t isolated immediately with the infant after delivery. According to Sylvia [11] et al. population-based retrospective cohort study, infants born to mothers with maternal TB had similar chance of preterm delivery compared to those born to mothers without TB. After adjusting women’s conditions to the same, the chance of preterm delivery was decreased but remained similar between infants born to mothers with and without TB, indicating that there is no association with prematurity.

TB may influence the function of reproductive organs which mainly affects fallopian tubes, next is endometrium and ovary, and therefore causes infertility [12]. IVF has become an effective therapy for a sterile woman after TB. The embryo growth rate and clinical pregnancy rate are chiefly dependent on the endometrial receptivity, which is an effect of the mutual coordination of ovarian steroid hormones, especially progesterone and estradiol [13]. For this patient, we preformed GnRH-antagonists protocol, which can induce a barrier of GnRH receptor with a rapid decrease in LH and FSH, preventing untimely LH surge and allowing the proper development of leading follicle. This protocol has a shorter duration of therapy and less gonadotropin usage, which may be a feasible alternative in an IVF cycle for a woman with infertility after regular antitubercular treatment.

In conclusion, disseminated TB has a significant impact on female fertility, but the diagnosis is difficult owing to its nonspecific clinical manifestations and incapacity to easily find miliary changes on a simple chest X-ray. Awareness of certain risk factors, such as vulnerability to exposure, those can impact the women in reproductive age is essential. More active efforts to diagnose and treat this disease are needed. Moreover, IVF treatment becomes a feasible approach for an infertile woman after TB.

## Data Availability

All the relevant data is included in the case report. Reasonable request for any additional data can contact with the corresponding author.

## Abbreviations

TB: Tuberculosis
IVF: In vitro fertilization
IVF-ET: In vitro fertilization-embryo transfer
CT: Computed tomography
MRI: Magnetic resonance imaging
PPD: Purified protein derivative
HMG: Human menopausal gonadotropin
OHSS: Ovarian hyperstimulation syndrome
